# Stem cell delivery of therapies for brain disorders

**DOI:** 10.1186/2001-1326-3-24

**Published:** 2014-07-19

**Authors:** Alexander Aleynik, Kevin M Gernavage, Yasmine SH Mourad, Lauren S Sherman, Katherine Liu, Yuriy A Gubenko, Pranela Rameshwar

**Affiliations:** 1 Graduate School of Biomedical Sciences, Texas, USA; 2 Department of Anesthesiology, New Jersey Medical School, Rutgers School of Biomedical Health Science, Newark, NJ 07103, USA; 3 Department of Medicine – Division of Hematology/Oncology, New Jersey Medical School, Rutgers School of Biomedical Health Science, Newark, NJ 07103, USA

**Keywords:** Mesenchymal stem cells, Glioblastoma, Neural stem cells, Therapy

## Abstract

The blood brain barrier (BBB) poses a problem to deliver drugs for brain malignancies and neurodegenerative disorders. Stem cells such as neural stem cells (NSCs) and mesenchymal stem cells (MSCs) can be used to delivery drugs or RNA to the brain. This use of methods to bypass the hurdles of delivering drugs across the BBB is particularly important for diseases with poor prognosis such as glioblastoma multiforme (GBM). Stem cell treatment to deliver drugs to neural tumors is currently in clinical trial. This method, albeit in the early phase, could be an advantage because stem cells can cross the BBB into the brain. MSCs are particularly interesting because to date, the experimental and clinical evidence showed ‘no alarm signal’ with regards to safety. Additionally, MSCs do not form tumors as other more primitive stem cells such as embryonic stem cells. More importantly, MSCs showed pathotropism by migrating to sites of tissue insult. Due to the ability of MSCs to be transplanted across allogeneic barrier, drug-engineered MSCs can be available as off-the-shelf cells for rapid transplantation. This review discusses the advantages and disadvantages of stem cells to deliver prodrugs, genes and RNA to treat neural disorders.

## Review

The BBB seems to be a major limitation for drugs to enter the brain. Stem cells can home to the brain. Thus, these cells can be the future form of treatment to bypass the BBB. At this time, any drug could be delivered with stem cells. The challenge will be to ensure that the stem cells can enter the brain without the eventual inflammation due to allogeneic responses. Since stem cells can respond to its microenvironment to undergo differentiation, this could be another challenge to ensure that after drug delivery the stem cells do not form a specialized cells within the brain. Since there are ongoing trials, it is presumed that the method would be improved for approved trials to treat any brain disorder.

## Introduction

The current standard of care for glioblastoma multiforme (GBM) uses temozolomide (TMZ)-based chemotherapy in conjunction with radiotherapy. Despite this type of aggressive treatment, patients are faced with high mortality, morbidity, cancer recurrence and short median survival. Current limitations in the treatment of GBM involve obstacles towards achieving complete tumor resection, difficulties in penetrating the blood brain barrier (BBB), insufficient accumulation of therapeutic agents at the site of the tumor, and tumor resistance. Thus, patients with GBM can benefit from alternative treatments with drug-loaded stem cells. Mesenchymal stem cells (MSCs) and neural stem cells (NSCs) have been shown to potential efficacious cells as alternative to drug delivery to GBM. Although this review discusses the use of stem cells for GBM, a similar approach can be applied for other neurological disorders. The approaches discussed in this review will depend on further studies to determine how the transplanted stem cells and the released therapeutic agents will affect normal brain functions.

### Neurodegeneration and neurotoxicity

A major setback for drugs intended for brain disorders is the inability to access the brain due to the surrounding BBB, which prevents crossing of large hydrophilic substances to the brain [1]. The nervous system has a vital role to send and receive electrochemical signals for organs to communicate. However, this communication can become dysfunctional as would occur in neurodegenerative diseases and brain injury. The importance of the brain in physiological responses makes it vital to develop therapies and more importantly, to identify how treatments such as stem cell-mediated delivery of drugs could be effective.

Neurotoxicity is a major limitation for the development of drugs that target the brain. A neural-targeted drug should not cause pathology such as changes in the expression of particular genes that could cause an increase in intracellular proteins. An understanding of these dysfunctions is needed to develop treatments for stem cell delivery to the brain.

Neural dysfunction in Alzheimer’s disease (AD) has been associated with the accumulations of hyperphosphorylated tau protein and amyloid beta aggregations, commonly referred to as neurofibrillary tangles and amyloid beta plaques, respectively [2]. The amyloid precursor protein (APP) can be cleaved by alpha or beta secretase. Under normal conditions, the precursor protein can be cleaved by alpha secretase into two subsequent strands, the soluble form of the precursor protein fragment and a membrane bound fragment (C83). Alternatively, the precursor protein may be cleaved by beta secretase, resulting in the production of a shorter soluble fragment (C99). In AD, it has been suggested that excessive amounts of the 40 and 42 amino acid peptides resulted in the latter aggregating to cause toxicity to surrounding neural tissue [3].

The accumulation of amyloid beta protein can be neurotrophic for undifferentiated hippocampal neurons and at high concentrations, neurotoxic to mature neurons [4]. The neurotoxicity is caused by axonal retraction of the differentiated neurons, resulting in cell death. Interestingly, a common region within the amyloid beta protein mediates show dual trophic and toxic effects. The small common region was reported to be antagonistic to the tachykinin peptides, which can be neurotransmitters.

In other types of dementia such as in Parkinson’s disease (PD), toxicity has been associated with Lewy bodies, which are abnormal aggregation of proteins that interfere with normal electrochemical signaling within the brain [5]. Alpha-synuclein has been suggested to be the primary protein associated with Lewy bodies and has been tied to mutations and/or over-expression of the gene [5].

Neural disorders are not always caused by neurodegenerative diseases. The brain can sustain physical injury and also, from the administration or exposure to chemicals. An example of physical injury is traumatic brain injury (TBI), which is of particular concern for athletes or victims of motor vehicle accidents. TBI could result in lasting deficits due to brain damage. Cerebral vascular accidents (CVA), otherwise known as stroke, can also occur in patients leaving them with focal neural deficits. However, the brain is also susceptible to various chemical substances, including those currently used in standard medical practice. Of particular focus are anesthetics.

Intranasal administration of the anesthetic isoflurane, has been suggested to decrease neurogenesis, primarily in the hippocampus and dentate gyrus in young Wistar rats [6]. *In vitro* studies suggested that particular doses of isoflurane do not induce death of NSCs but decreased their self-renewal property [6]. NSCs exposed to anesthetic expressed lower levels of the stem cell-associated gene, *Sox2*, suggesting evidence of NSC differentiation [6].

### Therapies - neural disease

The current methods to treat neurodegenerative diseases show little success. One of the main hurdles involves the delivery of efficacious levels of the drug to the central nervous system (CNS). The blood brain barrier (BBB) could be selective with regards to molecules, including drugs, crossing the barrier. Thus, there is a need to develop less invasive, but efficacious treatments that can bypass the hurdles associated with crossing the BBB to treat brain disorders.

The BBB regulates the passage of nutrients, ions, and other substances from the blood into the brain To cross the BBB effectively, the molecule should be less than 400 Da, lipid soluble with less than 8–10 hydrogen bonds with water as a solvent, and not be a substrate for an active efflux transporter at the BBB such as p-glycoprotein [7]. Efflux transport systems may target the drugs that meet these criteria and export them from the brain. As a result, the BBB excludes many small-molecules, and nearly all biopharmaceuticals such as gene and protein medicines, which fail to penetrate into the brain tissue to an appreciable extent [7]. To overcome the problem of delivering drugs to the brain, multiple strategies have been developed, as discussed in this review.

Memantine, a non-competitive antagonist to the *N*-methyl-D-aspartate receptor (NMDA) receptor, showed little benefit for AD [8]. Non-invasive procedures can be used for certain diseases such as hemorrhagic ischemic stroke [9]. Patients with ischemic stroke are usually treated with tissue plasminogen activator (tPA) to increase blood flow to the oxygen deprived brain region. This type of intervention could pose a great risk with regards to generalized bleeding [9]. In contrast, patients with TBI are given invasive treatment, although with little improvement [10].

In AD, the evidence indicated that reduced levels of the neurotrophic brain-derived neurotrophic factor (BDNF) could be attributed to the neurodegeneration. The administration of BDNF in experimental models of this neurodegenerative disorder showed that this could slow the process of neural degradation [11,12]. Due to the difficulty of crossing the BBB, the treatment with BDNF was delivered by intracranial injection. However, this method might not be practical for humans with neurodegenerative diseases. Other methods to deliver BDNF are under investigation. These include the delivery in BDNF in nanoparticles as well as ectopic expression of BDNF in stem cells. The application of stem cells to deliver drugs is discussed later in this review article.

### Non-stem cell approaches

The intravenous administration route and the BBB may be bypassed with intracerebral, intrathecal and intranasal route of delivery [13,14]. The BBB may be locally and reversibly disrupted to enhance the delivery of chemotherapeutic agents using microbubble facilitated focused ultrasound (MB-FUS) [15-18]. Passage of drugs through the blood brain barrier may be facilitated through chemical modifications to increase lipid solubility, plasma half-life, mask enzyme cleavage sites, and chemical conjugation strategies to target specific transport systems, receptor-mediated endocytosis/transcytosis, and adsorptive endocytosis/transcytosis [19,20]. The passage of drugs through the BBB may be improved through biological modifications of drugs involving the coupling of the drug to a receptor-targeted delivery vector such as a genetically engineered peptidomimetic monoclonal antibody [21-24]. Drug passage through the BBB may also be facilitated through novel strategies including encapsulation of therapeutic cells and their implantation into the surgical cavity, convection-enhanced drug delivery, direct perilesional injections, synthetic carriers such as metallic nanoparticles, and gene therapy [13,19,21,25-29].

**Figure 1:**
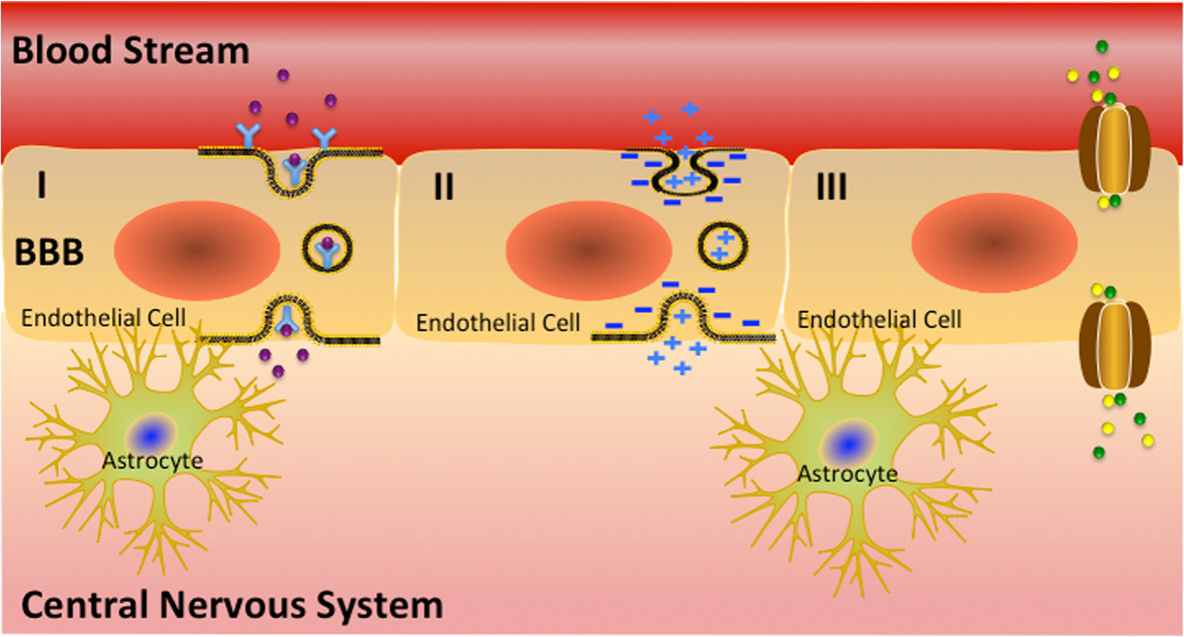
**Transport mechanisms across the blood brain barrier (BBB) are depicted. I)** Shows receptor-mediated transport, **II)** demonstrates non-specific uptake through adsorption-mediated transport, and **III)** displays carrier-mediated transport.

Invasive and non-invasive approaches were proposed to deliver drugs to the brain to treat GBM. Intracranial injection, intraventricular administration and the disruption of the BBB are examples of invasive delivery techniques used during neurosurgery. This method could however resulted in unwanted side effects such as disruption of the BBB, which is linked to chronic neuropathologic changes [30]. A gentler approach involves carrier- and receptor-mediated transport mechanisms using endogenous nutrient transport systems within the BBB (Figure 1). Carrier-mediated transport utilizes stereospecific molecular carriers or pores present on the apical and basolateral sides of the blood–brain barrier to carry small molecules such as ions, energy sources and amino acids [24]. Carrier-mediated transport systems can be used for noninvasive drug delivery by conjugating therapeutics to the natural substrates [24]. However, carrier-mediated transport will not work for large molecule drugs [7].

Receptor-mediated transport (RMT) mechanisms can be used to deliver larger molecules through the endogenous vesicular trafficking system of the brain endothelium such as the insulin, transferrin, or leptin receptor for brain influx of nutrients through a transcellular, receptor-mediated transport mechanism known as transcytosis [24]. Before therapeutics can be delivered through RMT, the drug must first be conjugated to a Molecular Trojan Horse (MTH)-a molecule that can target the natural RMT system [7]. RMT transport has significantly less size restrictions than carrier mediated transport due to a vesicle-based rather than a stereo selective pore-based delivery mechanism [7]. It is also important to note that RMT systems, which employ the vesicular trafficking machinery of the endothelium to transport substrates between blood and brain, have been proven to work *in vivo*[7].

The delivery of a neuropharmaceutical across the BBB through a receptor-mediated transport system requires linkage to the BBB delivery vector that targets a known blood-barrier receptor such as the transferrin receptor or the insulin receptor [31]. The neuropharmaceutical can be linked to the targeting vector either covalently or non-covalently and can be conjugated to its targeting vector through various methods including chemical linkage or non-covalent steptavidin/biotin linkage [32]. The highest priority for any conjugation strategy is to ensure that neither the transport vector nor the pharmaceutical product lose their function [32]. Chemical linkage strategies may involve linking the pharmaceutical drug to the targeting vector using lysine residues of either the targeting vector or the protein followed by chemical functionalization using Traut’s reagent, maleimide, and chemical spacer [33]. Non-covalent streptavidin/biotin linkage can involve monobiotinylation at lysine residues using N-hydroxysuccinimide (NHS) analogs of biotin, biotin hydrazide which reacts with carboxylic acid moieties on glutamate and aspartate residues, or through a thioester linkage between streptavidin and the targeting vector [34].

Alternative mechanisms for drug delivery through the BBB can involve liposomes and nanoparticles [28]. Liposomes can entrap water-soluble molecules in their aqueous core, absorb lipophilic drugs in their lipid bilayer membrane, complex with gene-based medicines, become more specific by coating with a BBB-targeting antibody, and become less resistant to removal by the reticuloendothelial system (RES) through the incorporation of PEG-disteraroyl-phosphatidylethanolamine (DPSE) moieties into the liposome bilayer [31]. Nanoparticles such as PBCA poly (butyl cyanoacrylate) can also be used to deliver neuropharmaceuticals to the brain by entrapping them in the nanoparticle matrix or attaching them to the surface of the nanoparticle [34].

### MSCs - cellular delivery of therapeutic agents

Stem cells are at the forefront in drug delivery, including the transfer of RNA to treat neurodegenerative disorders and brain tumors. Stem cells such as MSCs have been shown to cross the BBB [35,36]. This property provides stem cells with the advantage to overcome the current hurdles of delivering drugs to the brain. Despite a large number of methods to get drugs through the BBB, the method remains inefficient. MSCs are an intriguing delivery system to treat brain tumors such as GBM [37]. Once MSCs cross the BBB they can home to regions of tumor growth for targeted drug delivery [38]. MSCs could also serve as neuroprotectants [39]. In order to understand the benefit of MSCs, we first describe their basic biology, including their origin and immune functions.

The origin of MSCs in adults is a subject of debate. It is believed that MSCs are neuroectodermal. MSCs have shown ease with regards to differentiate into cells of all germ layers [40]. In general, the literature on stem cells is mainly focused on their ability to regenerate and protect tissues. There is relatively reduced emphasis on the ability of stem cells, such as MSCs, to be applied as cellular vehicle to deliver drugs across the BBB [41].

MSCs can be licensed to be immunosuppressor cells when they are placed within an inflammatory milieu and also when they are incubated with cytokines [42]. MSCs express chemokine receptors, which provide them with the ability to home to areas of tissue injury with a high level of chemokines [43]. It is unclear if a particular subset of MSCs has preference for homing to the sites of tissue injury. The selection of MSCs, based on the expression of chemokine receptor, could be an approach to select the population that shows preference for homing to the site of tissue insults and tissue degeneration [44]. Upon homing to the target site, MSCs can interact with the ligands found in the tissue for specific immune response. These types of responses are important for tissue repair and protection.

The pathotropic effects of stem cells to home to regions of tissue pathology underscores why the area of stem cell therapy are gaining attention for brain disorders. The use of stem cells to deliver drugs and RNA need to be tried for disorders that would otherwise have poor outcome [44]. Table 1 shows examples of clinical and experimental trails using MSCs.

**Table 1:** Clinical and experimental therapies with MSCs for neural-related diseases

**Diseases**	**Cells**	**Study**	**References**
Glioblastoma multiforme	MSC-Gene therapy	Experimental/Murine	[45]
Glioblastoma multiforme	MSCs: miRNA	Experimental/Murine	[46]
Glioblastoma multiforme	Gene therapy: (hsFlt3L/TK)	Experimental/Dog	[47]
Multiple Sclerosis	MSCs	Clinical Trial	NCT01883661
Experimental Autoimmune Encephalitis	MSCs	Experimental/Mouse	[48]
Parkinson’s Disease	MSCs	Clinical Trial	NCT01446614
Alzheimer’s disease	MSCs	Human	NCT01547689
Amyotrophic Lateral Sclerosis	MSCs	Experimental/Murine	[49]
Huntington’s Disease	MSCs	Experimental/Mouse	[50]

The table shows representative clinical and experimental trials with MSCs.

MSCs have advantages over other stem cells with regards to their use in cell therapy. MSCs can be easily expanded from tissues with little ethical concerns. While NSCs might be required to be obtained from autologous source, MSCs can be used across allogeneic barrier. Among the clinical trials studied, there is no evidence that MSCs can survive for prolonged period. There are other issues with MSCs when used to deliver drugs to brain cancer such as their supporting roles for tumors. This is a major issue and is therefore discussed in the next section.

### MSC therapy for brain pathologies

One area of study where MSC-based treatment has gained traction is for brain tumors, in particular gliomas, which represent the most common tumor of the brain. GBM is associated with low survival rates. It is difficult to treat GBM, partly due to the problems of transporting the drugs through the BBB. To overcome this hurdle, MSCs have been proposed as a method to use stem cells to deliver drugs for gliomas.

MSCs are especially useful for their pathotropism, which is their propensity to home at tumor sites. Stem cells loaded with carboxylesterase were used as a method to target tumor cells due to the ability of the enzyme to convert a prodrug into an active form [51]. An experimental study with rats genetically engineered MSCs to express “suicide genes” to target glioma. The engineered MSCs homed to glioma where they inhibited the glioma cell growth to improve the survival of the animal [52]. MSCs can be used to deliver miRNA mimics to target gliomas. MSCs transfected with miRNA-124 and miRNA-145 mimics inhibited the migration of glioma cells [53]. The aforementioned preclinical trials provided evidence that MSC-mediated delivery of therapeutic factors will soon be incorporated into clinical trials.

Other disorders can benefit from MSCs. Neurological disorders such as AD and multiple sclerosis have seen encouraging results from the research on MSC-mediated delivery of therapeutic agents. MSCs have been thought to promote remyelination in multiple sclerosis (MS), and to prevent degradation of existing myelin. In experimental studies, MSCs have proven to enhance remyelination when co-transplanted with oligodendrocyte precursor cells (OPC) [54]. The transplantation of OPCs alone was not sufficient to treat MS since these cells express alloantigens and could be subjected to immune rejection. To overcome immune rejection, OPCs could be co-transplantated with MSCs. The latter could thwart allorejection based on their immunosuppressive characteristics as well as their ability to home to the site of injury. The results showed that co-transplantation of MSCs increased the maturation of oligodendrocyte, myelination in the corpus callosum, and decreased the immune reaction from microglia and astrocytes.

MSCs can be ectopically expressed to secrete specific factors after homing to the brain for disorders such as MS. MSCs were genetically engineered to secrete IFN-β, which was shown to inhibit the effects of pro-inflammatory cytokines to ameliorate the symptoms of MS [55]. The preclinical successes have led to current studies to test the safety of autologous and allogeneic MSC transplantation, although most of those studies are in the recruiting stage.

The large number of preclinical trials indicated that MSC-mediated therapy show promise for cellular therapy. However, there are some concerns that have been expressed with the use of MSCs to deliver drugs, based on the disease. For example, in the case of gliomas, these are heterogeneous diseases. More importantly, there are noted differences in gliomas in rodent models and humans. There are concerns that these differences could impact clinical trials [56]. Most noted are the models of human GBM that are developed in immune deficient mice. Despite the route of injection intracranially, the microenvironment might be similar as the human brain, but the mice lacks an immune system. Histopathology of the experimental and human tumors showed significant differences. Another issue is that many studies injected GBM in the dorsal flanks of mice, which do not recapitulate GBM in human.

In addition to the questions regarding efficacy, there may also be concern for safety, as some studies have noted formation of tumors in MSC cultures from mice, and evidence of cell death due to inflammation and reactive oxygen species [57]. The rare reports of transformation reported for murine MSCs have not been noted for human. More importantly, MSCs can support the same tumors that they target for drug delivery [58]. Research studies are needed to determine how a suicide gene can be invoked to eliminate the MSCs after drug delivery.

Despite the possible side effects, there are several ongoing clinical trials with MSCs as a delivery method for the treatment of a number of brain pathologies including brain ischemia, Amyotrophic Lateral Sclerosis (ALS), MS, and AD. Studies have also shown that it may be possible to use MSCs for the delivery of therapeutic agents to gliomas through the release of exosomes [46]. Since exosomes are rich sources of miRNAs, which could be the future method of therapies, it is important to include a discussion on exosomes in this review article.

### Exosomes for miRNA delivery via stem cells

This section discusses the biogenesis of exosomes and the composition because their release could be used as a method to transfer small miRNA from MSCs to the brain tumors and also to treat neurodegenerative disorders. Indeed, RNA-loaded MSCs can deliver small RNA through gap junction and by their package in exosomes [46,59].

Exosomes or microvesicular bodies (MVBs) are membrane-bound nanoparticles, ranging between 30–100 nm in diameter [60]. The vesicles are at times considered as endosomes that fuse with the plasma membrane. Exosomes are released by various cell types such as immune cells, macrophages, lymphocytes, salivary gland epithelial cells, tumor cells, neurons, and MSCs [61,62]. The exosomes are small vesicle-like structures that transport proteins, lipids, mRNAs, and miRNAs [60]. Exosomes play an important role in intercellular communication and have been described to regulate immune functions, tumor migration and modulate the actions of drugs [32].

There is increasing evidence that exosomes are present in physiological fluids such as plasma and pleural effusions and urine from malignancy [63]. Exosomes have also been proposed as novel systems for the delivery of therapeutic agents [64]. An understanding of exosome biogenesis and composition will help to advance our understanding on the mechanisms by which MSCs are used in therapeutic applications.

Exosomes originate as endosomal intraluminal vesicles (ILVs) or MVBs that undergo endocytic fusion with the plasma membrane to release 50–90 nm intra-luminal vesicles into the extracellular milieu [65]. As an alternative fate, ILVs or MVBs can sequester proteins destined for degradation in lysosomes. The internal membranes of MVB contain cholesterol-rich membranes [66]. In addition, detergent-resistant membranes containing well-characterized raft marker proteins such as GPI-anchored proteins and flotillin have been isolated from the detergent resistant membranes of late endosomes [67]. These data suggested that lipid raft components in the endosomal system are sorted to the internal membranes of multivesicular bodies [68]. In proteomic and biochemical analyses of exosomes, an enrichment of several raft proteins and lipids was observed [68]. These observations suggested a connection between MVB and immune responses [68]. In fact, it appears that HIV budding in primary macrophages occurs through the exosome release pathway [68]. By budding through lipid rafts in T-cells, HIV selectively incorporates raft markers and excludes non-raft proteins. These findings are in agreement with results that indicate the involvement of rafts in the interaction of HIV with host cells [68].

### MiRNAs

The influence of exosomes on the stem cell microenvironment and ensuing cellular function has also been studied within the context of miRNA. The exosome-containing miRNAs can modulate functional changes when transferred to cells. MiRNAs are small (18–24 nucleotides) endogenous non-coding RNAs that act as post-transcriptional regulators of gene expression and thus play crucial roles in regulating multiple cellular processes [69]. MiRNAs are first synthesized as primary transcripts, referred as pre-miRNAs [69]. These structures are then processed as pre-miRNAs by the nuclear protein DGCR8 and the ribonuclease Drosha before exported to the cytoplasm via exportin-5 [70]. Pre-miRNAs are further modified by the RNase III enzyme Dicer to form mature double-stranded miRNA duplexes [55,56]. Argonaute proteins then associate with these duplexes to create the miRNA-induced silencing complex (miRISC) that acts as the machinery by which miRNAs can regulate the translation of transcript targets.

MiRNAs can cause degradation or translational repression of mRNA to negatively regulate more than 30% of the genes to control cell proliferation, inflammation, and metabolism. Thus, miRNA could be involved in the deregulation of key signaling cascades to disrupt cellular homeostasis and induce carcinogenesis, such as GBM [71]. The biological functions of miRNAs are highly significant to the treatment of brain disorders.

MiRNAs are involved in the development and progression of brain tumor [46,71]. There are several reports on the literature on miRNAs in tumor development. Similar to most pathological system, the role of miRNAs is complex. On a more general function, miRNAs are involved in the regulation of genes with oncogenic and tumor-suppressive properties [72].

Studies on miRNA in GBM and other neurodegenerative disorders are important to future therapy with RNA. This would require efficient delivery of RNA across the BBB. As examples, miRNAs and antagomiRs can target specific molecules within the signaling pathways caused by the activation of specific receptors within the disease. In the case of GBM, the receptor tyrosine kinase epidermal growth factor receptor (EGFR) is activated to promote cell growth, resistance to apoptosis, adhesion and cell migration [73]. MiRNAs inhibited the signaling of EGFR in GBM by blunting the signaling networks involving PI3/Akt pathway [74]. This resulted in chemosensitization of the frontline treatment for GBM, TMZ. MiRNAs might function at multiple levels since miR-21 and miR-26 appeared to be involved in the increased expression of EGFR and decreased the tumor suppressor gene, *PTEN*[75,76]. EGFR is overexpressed in 40-60% of primary GBM tumors and this subset of GBM results in worst prognosis [73,75]. Thus, the identification and efficient delivery of miRNAs would be significant for EGFR-expressing GBM.

MiRNAs can also prevent GBM progression and development by differentially regulating the expression of key genes in brain tumors. MiR-218 and miR-326 have been reported to decrease IκB kinase thereby inhibiting the activation of NF-κB [77]. MiR-326 has been shown to regulate the Notch signaling in GBM [78]. The evolutionary conserved miR-9 could also be targeted. However, caution has to be exerted due to its additional neuroprotective function [46,79]. Table 2 outlines how representative miRNAs can regulate key signaling molecules, suggesting potential targets for GBM. Regardless of the identification of targets for GBM or other brain pathologies, the delivery of miRNA and other therapeutics must first have to bypass the blood brain barrier [25,79]. This issue is discussed throughout the text since it is fundamental to brain therapies.

**Table 2:** MiRNAs and key molecules in signaling pathways reported for gliobastoma multiforme

**Target**	**miRNA**	**Pathway/Process**	**References**
EGFR	miR-7	EGFR signaling	[80]
PTEN	miR-21	Cell-cycle progression, Apoptosis	[75]
PTEN	miR-26a	Akt pathway	[76]
Notch1/Notch2	miR-326	Notch pathway	[78]
IκB	miR-218	IKK-β/NF-κB pathway	[77]
STAT1/STAT2	miR-221/miR-222	IFN-α signaling pathway	[81]
CDK6	miR-124/miR-137	Cell cycle arrest	[82]
Bmi-1	miR-128	Bmi-1 decrease	[83]
Akt1, CyclinD1, MMP-2, MMP-9, Bcl-2	miR-451	Tumor suppression	[84]

Table shows representative examples of how specific molecules are regulated by miRNAs, and to provide insights on how the information can be used to develop treatment strategies.

Overall, the discussion in this section when combined with the previous section on exosomes, showed that exosomes could be designed with the intent to deliver specific miRNA. A method by which this could occur is to express the desired miRNA in MSCs. The miRNAs can then be packaged as exosomes. The MSCs, which can cross the BBB, would then release exosomes within the brain. This method is advantageous as it will bypass the BBB to deliver small RNA to the brain. The method could be therapeutic in altering biological processes [60].

### Stem cell as a vehicle of drug delivery

Although discussed above, this section revisits the use of MSCs and discusses the advantages of these stem cells for drug delivery. MSCs are inherently immunosuppressive and have the ability to home to a variety of primary and metastatic tumors including breast, colon, ovarian, lung carcinomas, as well as gliomas [38]. An examination of the clinical trial registry (clinicaltrials.gov) indicates that MSCs are prominent for drug delivery in cancers, including ovarian, prostate, head and neck as well as hematological malignancies. MSCs can be expressed to deliver cytokines, prodrugs, apoptosis inducing proteins, and anti-angiogenic agents [85-92]. These findings have to be considered with the caveat that MSCs are also capable of exerting pro-tumorigenic effects and that MSCs should be reliably tracked once administered [38].

MSC delivery of therapeutic agents can overcome obstacles related to anatomical barriers, drug half-life, and tumor targeting [86]. Cytokines with anti-tumor properties such as IL-2, IFN-β, and tumor necrosis factor-related apoptosis-inducing ligand (TRAIL) have limited utility due to a short half-life *in vivo* and their toxic effect on normal cells [93]. A major advantage for using MSC as cytokine delivery vehicles is the ability of MSCs to selectively migrate to the tumor site and release the intended agent [93]. In addition to MSCs selectively homing to solid tumors they also actively home to sites of metastases, distant from the primary tumor [33].

**Figure 2:**
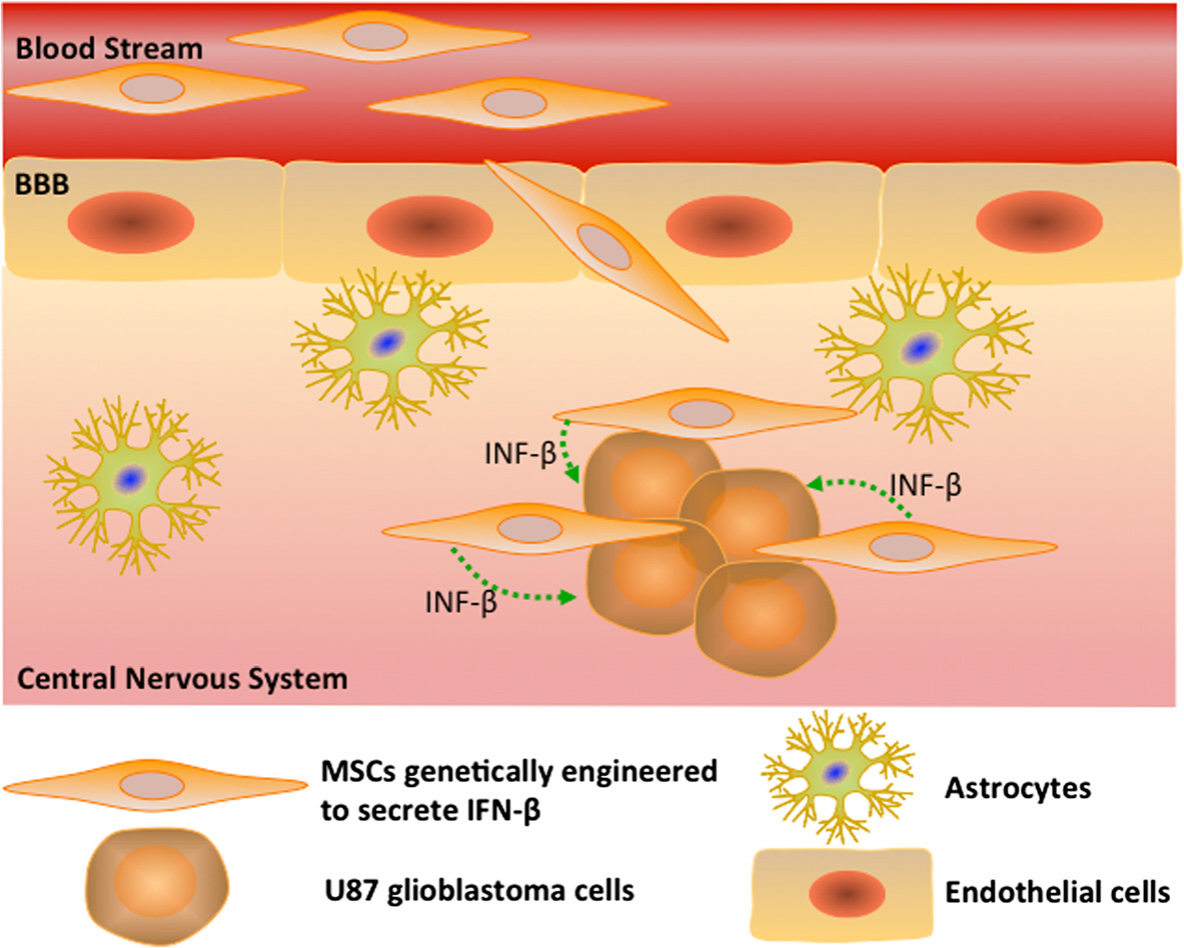
**MSCs genetically engineered to secrete IFN-β injected into the rat internal carotid artery can penetrate the BBB and home to U87 glioblastoma cells within the central nervous system (CNS).** Administration of these genetically engineered MSCs through the rat’s tail vein or subcutaneously does not lead to penetration of the BBB or MSC tumor homing. This tropic mechanism is mediated by several receptor ligand combinations and is correlated with bulk tumor size reduction.

Due to the problems of getting drugs through the BBB, GBM could be an ideal cancer for MSCs in drug delivery [24,25]. Despite decades of research and advances in the treatment of this disease with surgery, radiotherapy, and chemotherapy, there is no cure and the current prognosis gives a median survival of only 12–18 months [25,33]. Current therapies fail to cure the disease due to the inability to selectively target tumor cells, which have disseminated into the normal parenchyma of the brain, at sites distant from the tumor mass [25]. MSCs have the potential to provide neurotrophic support and deliver therapeutics to sites of brain pathology after intra-arterial administration, implantation directly into the tumor bed, implantation at a distant intracranial site from the tumor bed in the same hemisphere or collateral hemisphere, implantation into brain ventricles, or implantation into a peripheral intravascular site as confirmed by studies in rodents [94,95]. In a recent experimental study with GBM, MSCs have been shown to home to the solid tumor through tropic mechanisms, as outlined in Figure 2[95]. This tropism could be mediated by several receptor-ligand combinations, which is correlated with reduced tumor burden [95]. While the migratory properties of MSCs are intriguing, it is also important to note that MSC-based treatments could also support tumor growth [38,96].

## Conclusion

MSCs have the potential as cellular vehicles for drugs and other molecules to treat patients with neural diseases such as GBM, AD, PD, TBI and other neuropathologies for which limited treatment options exist. When considering the limitations of current methods of drug delivery to the brain, MSCs have the potential to become a safe cellular delivery vehicle containing a prodrug as well as ectopically expressed genes for targeted delivery. The affinity for MSCs to migrate to the brain combined with the relative ease for expanded MSCs make them attractive for gene and drug delivery.

## Abbreviations

AD: Alzheimer’s disease; ALS: Amyotrophic lateral sclerosis; APP: Amyloid precursor protein; BBB: Blood brain barrier; BDNF: Brain-derived neurotrophic factor; CVA: Cerebral vascular accidents; CNS: Central nervous system; GBM: Glioblastoma multiforme; GPI: Glycophosphatidylinositol; ILVs: Intraluminal vesicles; MSCs: Mesenchymal stem cells; MS: Multiple Sclerosis; MVBs: Microvesicular bodies; NSCs: Neural stem cells; NMDA: *N*-methyl-D-aspartate receptor; OPC: Oligodendrocyte precursor cells; PD: Parkinson’s disease; RES: Reticuloendothelial system; RMT: Receptor-mediated transport; TBI: Traumatic brain injury; TRAIL: Tumor necrosis factor-related apoptosis-inducing ligand.

## Competing interests

The authors declare that they have no competing interests.

## Authors’ contributions

AA wrote and edited the article. KMG and YSHM wrote parts of each section of the article. LS wrote parts of the article and prepared the figures. KL edited the entire article. YAG wrote and edited the sections on anesthesiology. PR formed the concept of the article, wrote sections in all parts of the review and edited the final manuscript. All authors read and approved the final manuscript.
